# Dissertation sur la Rage, qui a remporté le premier prix de la Société Royale de Medecine de Paris, le 11 Mars, 1783

**Published:** 1786

**Authors:** M. Le Roux

**Affiliations:** Chirurgien Major de l'Hopital General de Dijon, Associé de l'Academie Royale des Sciences, Arts et Belles Lettres de la méme Ville, et Correspondant de la Société Royale de Medecine de Paris


XIX *Dissertation sur la Rage, qui a remporté le premier prix de la Société Royale de Medecine de Paris, le* 11 *Mars,* 1783. *Par M.* Le Roux, *Chirurgien Major de l'Hopital General de Dijon, Associé de l'Academie Royale des Sciences, Arts et Belles Lettres de la même Ville, et Correspondant de la Société Royale de Medecine de Paris;* 4to Paris.

THIS dissertation on the hydrophobia, which obtained the prize offered by the Royal Medical Society at Paris, for the best essay effay on that fubject, feems to be the production of a candid and judicious writer, who has had opportunities of feeing and treating this dreadful diforder.

Diffections, M. le Roux very juftly obferves, have hitherto afforded us no fatisfactory information concerning the nature of this complaint.

In the three cafes he has feen, and which are related in the work before us, the patients were ill five or fix days before the dread of water took place; fo that he thinks we may very properly diftinguifh two periods in this difeafe; viz. that which precedes, and that which follows the appearance of this fymptom. In the three cafes in queftion, the duration of the firft of thefe periods was from five to fix, and that of the latter from three to four days.

M. Le Roux thinks, with the generality of the later writers on this fubject, that the difeafe is conftantly announced by fome alteration in the part where the wound was inflicted *. This opinion has lately been controverted by an ingenious and well-informed phyfician, who acknowledges, however, that in two of the

* See Vol. 3. p. 93. and Vol. 5. p. 85.

three three cafes he has defcribed*, pain in the limb bitten was amongft the firft fymptoms which preceded the hydrophobia; and that in the third, where the cheek was wounded, pain and tightnefs about the temples accompanied the commencement of the diforder, although in neither of the cafes did the wounded part undergo any change.

In a work intitled *Obfervations fur la Rage*, printed in 1780, M. Le Roux had adopted the opinion of Sauvages, and others, that when the virus contained in the wound begins to act, it is carried into the circulation; but he now thinks, that the phenomena of hydrophobia, may be more eafily and fatisfactorily accounted for by attributing them to a local nervous irritation; and he contends that the falivation which takes place in thefe cafes, the fenfe of fuffocation, the tightnefs acrofs the throat, the dread of water, and other fymptons, may be as eafily conceived to arife from fuch a caufe, as thofe of tetanus may from an external wound; at any rate, whether the blood is infected or not, by abforption, in thefe cafes, he

* Cafes and obfervations on the Hydrophobia: by J. Vaughan, M. D.

agrees agrees in opinion with thofe writers who think, that the virus remains in the wound till the firft period of the difeafe, and of courfe, that its effects may be prevented from taking place, by deftroying the texture of the part at any time between the infliction of the wound and that period. His fole reliance is on this mode of prevention, as he is convinced that all the internal remedies (and he enters into an ample difcuffion of each) that have been hitherto recommended, either as prophylactic or curative, in this melancholy difeafe, are abfolutely inefficacious.

He fuppofes the cafe of Elizabeth Bryant, defcribed by Dr. Nugent, in his Effay on the Hydrophobia, as having been cured of that difeafe, to have been merely an hyfterical affection *.

* Dr. Hamilton, in a work lately publifhed on this fubject, and which the reader will find more particularly referred to in the next article, adopts a fimilar opinion of the cafe related by Dr. Nugent: and is alfo inclined to think, that in the cafe defcribed by Mr. Wrightfon, in the Medical Tranfactions, Vol. ii. as a fuppofed cure of canine madnefs, the fymptoms proceeded entirely from fear.

The following is the mode of cauterizing the wound, recommended by M. le Roux, in theſe caſes.

The depth and extent of the bite are firſt to be carefully aſcertained, and the wound is then to be dilated with a biſtoury throughout its whole circumference, and in different directions, ſo that the orifice may be larger than the bottom, and that every part of the wound may be brought to view.

After the wound has been ſuffered to bleed, it is to be waſhed with ſoap and water, and dreſſed with dry lint: the day following, when the firſt dreſſing is removed, every part of the wound, and even the adjacent ſkin, is to be touched with a wooden probe, moiſtened with butter of antimony, in a ſtate of deliqueſcence. All the parts touched with this application inſtantly become white, and are ſometimes burnt to a conſiderable depth.

The wound is next to be covered with a large bliſter, and when this is removed, proper dreſſings are to be applied to the wound till the eſchar comes away, which generally happens on the ſixth or ſeventh day.

The cauſtic and bliſter are to be repeated in this manner at different times, as ſoon as granulations of flefh begin to appear in the wound; and finally, one or more iffue peafe are to be placed in the ulcer fo as to keep it open at leaft forty days.

As a reafon for not applying the butter of antimony at the firft dreffing, the author obferves, that the blood decompofes and deprives it, in a great meafure, of its corrofive quality. We cannot but think, however, that in cafes of this fort not a moment fhould be loft.

As proofs of the efficacy of this mode of prevention, the author mentions the cafes of eleven perfons who were bit by a mad wolf on the 14th and 15th of March, 1780. Of thefe, two remained at home and died of hydrophobia; of the cafes of the nine others, who were under the author's care in the hofpital at Dijon, the following is an abridged account:

1. A youth aged feventeen, was admitted March 17th, with feveral large lacerated wounds on his head, face, and right arm; and difcharged quite well on the 18th of May.

2. A boy, ten years old, was admitted on the 19th of March, with feveral large wounds on his head and face, and difcharged well on the 27th of May.

3. A

3. A girl aged two years, was admitted on the 20th of March, with a confiderable wound quite through her left cheek, and other wounds in different parts of her face: fhe was fent home well on the 27th of May.

4. A boy five years old, was admitted on the 20th of March, with feveral lacerated wounds on his head and face; one of thefe penetrated through the under lid of the right eye into the orbit, and was not cauterized. Thefe wounds were treated in the manner above defcribed, and all had been healed a fortnight, when on the 6th of May, the boy began to complain of pain in his right eyelid, the cicatrix of which appeared inflamed, and the day following it difcharged a bloody ichor. Symptoms of fever now followed, and on the 12th of May, a dread of water, at intervals. This laft fymptom was more confirmed on the 13th, and the child died in the night of the 16th of May. In this cafe the author remarks, no difcolouration took place, nor did the patient complain of pain previoufly to the attack of hydrophobia, in any of the wounds that had been cauterized.

5. A man aged twenty-eight years, was admitted on the 23d of March, with almoſt the whole of his lower lip torn off, and ſeveral other large wounds in different parts of his head and face, ſome of which were nearly healed. This patient died of hydrophobia on the 27th of May.

6. A girl twelve years old, who had been thrown down by the wolf, received two contuſions and ſeveral ſcratches on her boſom: theſe were cauterized, and ſhe experienced no ill effects from the accident; but there ſeems much reaſon to doubt, whether the contuſions, &c. were inflicted by the teeth or the claws of the animal; ſo that this caſe can have but little weight.

In the 7th, 8th, and 9th caſes, the patients were bit through their clothes; and we learn that theſe, as well as the ſubjects of the 1ſt, 2d, 3d, and 6th caſes, were in perfect health in January 1782.

M. le Roux compares the reſults of theſe caſes with thoſe of fifteen caſes at Senlis, in which mercury was adminiſtered as a prophylactic, lactic, and draws conclusions favourable to his own method; but we fear that a much greater number of facts will be requisite before the efficacy of this or of any other mode of prevention can be satisfactorily established.

It has fortunately been proved by repeated experience, that of the persons who are bit by a mad animal, only a very small number feel any bad effects from the accident, whether means of prevention are adopted or not; and on the other hand, we have to lament that cases have occurred, in which even the caustic, though applied almost immediately after the bite, has failed to produce the wished-for effect. Of this we have a melancholy instance in the case which is the subject of the following article.

